# Treatment Options for Motor and Non-Motor Symptoms of Parkinson’s Disease

**DOI:** 10.3390/biom11040612

**Published:** 2021-04-20

**Authors:** Frank C. Church

**Affiliations:** Department of Pathology and Laboratory Medicine, The University of North Carolina School of Medicine, University of North Carolina at Chapel Hill, Chapel Hill, NC 27599, USA; fchurch@med.unc.edu

**Keywords:** Parkinson’s disease, neurodegenerative disorder, substantia nigra, carbidopa/levodopa, anti-inflammatory, antioxidants, integrative medicine, older adults, motor and non-motor symptoms

## Abstract

Parkinson’s disease (PD) usually presents in older adults and typically has both motor and non-motor dysfunctions. PD is a progressive neurodegenerative disorder resulting from dopaminergic neuronal cell loss in the mid-brain substantia nigra pars compacta region. Outlined here is an integrative medicine and health strategy that highlights five treatment options for people with Parkinson’s (PwP): rehabilitate, therapy, restorative, maintenance, and surgery. Rehabilitating begins following the diagnosis and throughout any additional treatment processes, especially vis-à-vis consulting with physical, occupational, and/or speech pathology therapist(s). Therapy uses daily administration of either the dopamine precursor levodopa (with carbidopa) or a dopamine agonist, compounds that preserve residual dopamine, and other specific motor/non-motor-related compounds. Restorative uses strenuous aerobic exercise programs that can be neuroprotective. Maintenance uses complementary and alternative medicine substances that potentially support and protect the brain microenvironment. Finally, surgery, including deep brain stimulation, is pursued when PwP fail to respond positively to other treatment options. There is currently no cure for PD. In conclusion, the best strategy for treating PD is to hope to slow disorder progression and strive to achieve stability with neuroprotection. The ultimate goal of any management program is to improve the quality-of-life for a person with Parkinson’s disease.

## 1. Introduction

It is estimated that one million people in the United States are living with Parkinson’s disease (PD), with approximately 60,000 new cases diagnosed nationally each year [1,2,3,4,5]. The global prevalence of PD is believed to be up to 10 million people. PD symptoms occur due to the progressive loss of dopamine-producing neurons in the substantia nigra pars compacta region of the brain. Symptoms typically occur gradually over several years, making diagnosis challenging [5]. PD is traditionally characterized as a motor system disorder with four cardinal symptoms: bradykinesia (slowness of movement); rigidity (stiffness of the limbs and trunk); postural instability (impaired balance and coordination); and tremor (trembling in hands, arms, legs, and face) [6,7,8,9]. Though not as visible as these motor symptoms, non-motor symptoms are also experienced by many PwP as a part of their disease. The most common non-motor symptoms of PD include constipation, urinary dysfunction, depression, psychosis, apathy, and sleep disorders [9,10,11,12].

PD occurs most commonly in people aged over 60 years old [5]. In this group, most cases of PD occur sporadically and due to etiologies including neuroinflammation and oxidative stress, dysfunction of the innate and/or adaptive immune systems, mitochondrial activity disruption, genetic mutation, intracellular protein denaturation and aggregation, and environmental factors [1,2,3,4,5]. Interestingly, cases of PD in younger people are usually linked to particular genotypes [13]. At present, PD remains an incurable disease. As such, treatment goals in PD management center on slowing or halting disease progression [1,14]. The complexity of the factors that contribute to the development of the major sporadic form of PD demands a multi-pronged therapeutic intervention and plan to halt or slow PD progression. Accordingly, this review aims to describe a comprehensive and integrative treatment protocol for PD.

## 2. Treatment Plan for PD

The traditional approach for treating PD typically begins with a pharmacologic dopamine replacement strategy [1,5,14,15]. The first line for such therapy is either daily oral carbidopa/levodopa or a dopamine agonist. Some drugs prolong the lifetime of endogenous dopamine. Either along with or alternative to dopamine replacement, complementary and alternative medicine (CAM) and integrative medicine approaches are used by many to improve brain and overall health in PwP [16,17,18,19,20,21]. Lifestyle modifications can provide therapeutic benefits, as different forms of strenuous aerobic exercise are neuroprotective, on top of the general quality-of-life (QoL) benefits offered by regular exercise [10,12,22,23,24,25,26,27,28,29,30,31,32,33,34,35,36,37,38,39,40,41,42]. PD is a complicated disorder such that two PwP might have different symptoms with varied rates of progression and likely follow different treatment strategies, despite diagnosis with the same disease. Non-motor symptoms are prevalent in PD. Thus, PwP must communicate clearly with their healthcare team to address these issues and treat PD’s motor and non-motor symptoms.

**Figure 1:**
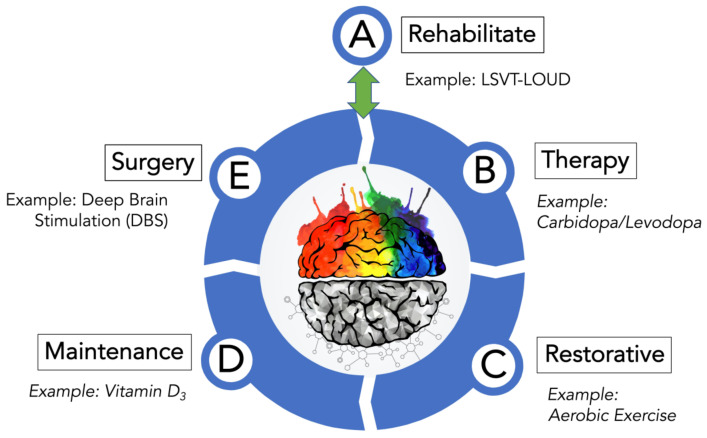
Treatment options for PD. As depicted, this PD-directed integrative and health strategy features five areas where intervention can be used to manage the numerous symptoms of PD. A treatment example from each category is given. Although drawn as a stepwise progression from A–E, this is not suggesting that PwP engage all five treatment options at all or in order of their presentation (Figure 1). Treatment option A (Rehabilitate) is considered a reversible entry and exit point to the treatment wheel described by options B (Therapy), C (Restorative), D (Maintenance), and E (Surgery). Please note that the various treatment options are not isolated silos that can only be accessed after fulfilling the prior treatment option. It is more likely that PwP, with guidance and advice from movement disorder neurologists, would likely use several components found in treatment options A–D that may change as their disorder progresses before possibly moving finally to option E (Surgery). Post-surgery-PD patients, assuming a successful response to surgery and under continual guidance from their medical team, would re-engage the A–D treatment options. Furthermore, aspects of the Therapy, Restorative, and Maintenance treatment options overlap and complement one another to develop an effective PD treatment plan.

The treatment plan described here incorporates these aforementioned traditional and non-traditional approaches that could help keep PD from progressing. The treatment strategy complements these options with an initial rehabilitation program to carefully assess and address PD before beginning other treatment options and with surgery, in cases where PD has resisted management by the other treatment options. Each of the five steps of this treatment plan for PD offers a comprehensive healthcare strategy and therapy that has been studied or found to be effective in either human or rodent animal studies. Described here is an integrative medicine and health strategy for PD that features five treatment options: rehabilitate, therapy, restorative, maintenance, and surgery (Figure 1).

### 2.1. Rehabilitate Options for Treating PD

Before PwP begin pharmacological therapy for PD, movement disorder specialists are likely to recommend PwP visit a physical therapist, occupational therapist, or a speech pathology therapist. The goal of such consultation would be to begin management of some of the altered motor symptoms. The reduction of dopamine in PD typically softens the voice and limits the body movements of PwP. Two programs called LSVT-LOUD [43,44,45] and LSVT-BIG [31,46,47,48] are directly targeted to helping the PwP speak louder and make larger movements, respectively.

The majority of PwP have speech/voice dysfunction negatively impact communication. LSVT (Lee Silverman Voice Treatment)-LOUD enhances the voice, increases vocal loudness (by improving articulation, vocal quality, and intonation), and positively alters PwP functional skills communication [43,44,45]. PwP also typically have a movement that is slow (bradykinesia) and hesitant (akinesia) with smaller amplitude (hypokinesia). LSVT-BIG uses intensive exercises of large-amplitude movements to overcome bradykinesia and hypokinesia in PwP [31,46,47,48]. Furthermore, LSVT-BIG yields movement focused on amplitude, resulting in bigger, faster, and increased movement precision.

LSVT-LOUD and LSVT-BIG require a neurologist trained to administer the programs, as are the physical therapist (LSVT-BIG) and speech pathology therapist (LSVT-LOUD) certified to oversee them. Both programs are for one hour per day, four days/week, for a total of four weeks. Afterwards, PwP can use these exercises from each program to continue on their own. While there are other programs that provide similar assistance, LSVT-LOUD [43,44,45] and LSVT-BIG [31,46,47,48] pioneered these programs specifically to help rehabilitate PwP [49].

An essential feature of rehabilitation is regular exercise. As mentioned above, many of the motor defects associated with PD cause stiffness, impaired balance, and slow movement. Under the ongoing guidance of their movement disorder neurologists, PwP should develop a regular exercise routine that incorporates stretching, movement, strength training, and aerobic exercise. There are many well-trained physical therapists with expertise in exercise-specific routines for PD. There are also numerous exercise modalities that PwP use to improve their QoL, including PWR!Moves, Rock Steady Boxing, and Dance for PD programs, power walking with poles, stationary biking, tai chi, and yoga [8,16,22,24,50,51,52]. Thus, rehabilitation therapy should be utilized throughout all stages of the disorder.

### 2.2. Therapy Options for Treating Motor Symptoms of PD

**Figure 2:**
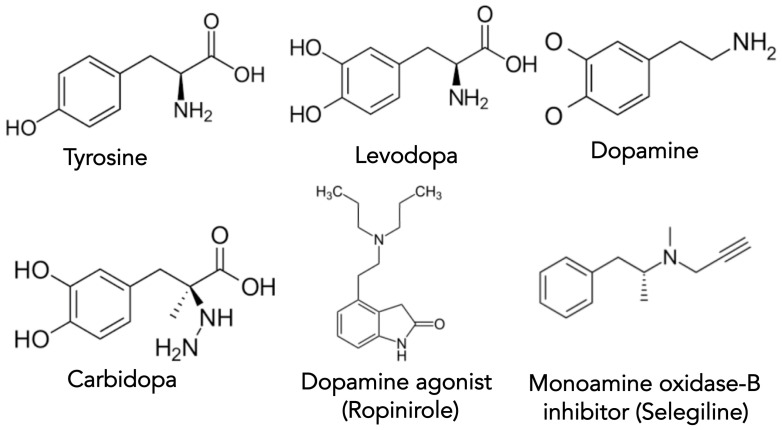
Structures of some of the key molecules involved in dopamine synthesis, drug therapy, and PD.

Dopamine has the chemical structure of 3,4-dihydroxyphenethylamine and is a member of the catecholamine and phenethylamine molecular families [53]. Like other neurotransmitters, dopamine delivers messages throughout the central nervous system (CNS) [54]. Dopamine is a derivative of the amino acid tyrosine (Tyr), where the enzyme tyrosine hydroxylase converts Tyr to levodopa (DOPA) [53,54]. From there, DOPA decarboxylase removes carbon dioxide from DOPA to produce dopamine [53,54]. The structures of dopamine and its precursors and some dopamine-regulating therapeutics are shown in Figure 2.

Table 1 gives a list of the majority of drugs approved by the FDA that are available to treat the motor symptoms of PD [55]. As PD is first and foremost a disorder of dopamine deficiency, dopamine replacement remains the standard therapeutic aim [56]. The combination of levodopa with carbidopa, an aromatic l-amino acid decarboxylase inhibitor, provides the most significant amount of symptomatic relief with the least adverse side-effects in treating PD [57,58]. The addition of carbidopa prevents the conversion of levodopa (i.e., DOPA) to dopamine in peripheral tissues, allowing for a successful transport of levodopa to the CNS [14]. Interestingly, the blood-brain barrier allows levodopa access into the CNS but denies entry to both dopamine and carbidopa (compare molecular structural differences in Figure 2). There are multiple formulations for carbidopa/levodopa tablets (Table 1). Alternatively, Duodopa is a continuously infused intrajejunal gel of carbidopa/levodopa [59]. Additionally, subcutaneous infusion of carbidopa/levodopa is under evaluation [60,61]. The major side-effects of carbidopa/levodopa are the development over time of dyskinesia and fluctuating ‘off-on’ periods of effectiveness [5]. The potential neurotoxicity of carbidopa/levodopa has been suggested [62]. However, Ahlskog recently reviewed and refuted the evidence that carbidopa/levodopa is neurotoxic [63].

Dopamine agonists mimic dopamine by binding to dopamine receptors in the CNS. They are used as a monotherapy for many PwP early in the treatment of their disease [64]. The original drug used to treat PD is apomorphine injection, a dopamine agonist, which is comparable in effect to levodopa but it has a shorter duration time [65]. Dopamine agonists are also frequently paired in combination with carbidopa/levodopa, especially as a “bridge” to stabilize the on–off periods PwP may experience on long-term carbidopa/levodopa therapy [1,14]. There are multiple dopamine agonists with both immediate- and extended-release forms (Table 1). There are several troubling side-effects that a minority of PwP encounter with dopamine agonists, mostly centered around impulse control disorders (e.g., pathologic gambling, shopping, internet use, and hypersexuality) [1,14].

Monoamine oxidase B (MAO-B) inhibitors are substances that inactivate the enzyme responsible for the inactivation of dopamine [66]. The MAO-B inhibitors Selegiline and Safinamide are used adjunctively with carbidopa/levodopa while Rasagiline is used either as monotherapy or in concert with carbidopa/levodopa (Table 1) [5]. MAO-B inhibitors may provide relief from symptoms as they help regulate the degradation of dopamine in peripheral tissue, which leads to increased half-life and availability of levodopa in the CNS. The SELEDO (from Selegiline plus Levodopa) study was a 5-year trial to assess the potential advantage of combining Selegiline and Levodopa in PD [67]. In treating early-stage PD, the combination of Selegiline and Levodopa was better than Levodopa alone.

Inhibitors of cathecol-*O*-methyl transferase (COMT) enzymes prevent the processing of levodopa to 3-*O*-methyldopa [68,69]. COMT inhibitors increase the half-life of levodopa, allowing more levodopa remain in the patient’s CNS for a longer period of time. Similar in concept to the MAO-B inhibitors but different in mechanism, COMT inhibitors preserve levodopa in PwP experiencing motor fluctuations with carbidopa/levodopa therapy. Similar to MAO-B inhibitors, COMT inhibitors can be used adjunctively with carbidopa/levodopa [1,5].

The history behind the use of Amantadine in PD is fascinating [70,71]. Amantadine was made and used initially as an anti-influenza medication. It turns out that PwP taking Amantadine to prevent the flu showed better control over their tremor. Amantadine provides help with most PD motor symptoms and it might be useful in PwP who have a prominent tremor or levodopa-induced dyskinesia.

### 2.3. Therapy Options for Treating Non-Motor Symptoms of PD

PD can also be considered a neuropsychiatric disorder [72]. Several neuropsychiatric symptoms are related to emotional and cognitive problems [73]. The neuropsychiatric symptoms are a significant disruption that contributes to disability in PwP [74,75]. There are symptoms related to the disease itself, including apathy, depression, and anxiety [76,77]. These non-motor symptoms are frequently present in the earliest PD stages, even preceding the origination of the motor symptoms. This suggests that both non-motor and motor-related symptoms of PD are associated with reduced dopaminergic production.

The second type of PD neuropsychiatric symptoms exists as a side effect of dopaminergic replacement therapy [73,78]. The impact of the medication can result in addiction, hypomania, nocturnal hyperactivity, and punding [73,78]. Managing the non-motor symptoms of PD presents a challenge to the physician because they must differentiate the contribution from medication, disorder progression, and the PD patient’s emotional state.

Table 2 lists most of the drugs approved by the FDA that are available to treat the non-motor symptoms of PD related to depression and anxiety, excessive drooling, and gastrointestinal problems [79,80,81,82,83]. Depression occurs in up to 50% of PwP at some point during the disorder [84]. PwP with depression are typically treated with a standard antidepressant from the class of SSRIs, SNRIs, and other similar neurotransmitter reuptake inhibitors (Table 2). Anxiety in PD takes many forms but is generally described as feelings of worry, panic, unease, and jitteriness [84]. Besides psychotherapy, medication options include SSRIs, and Buspirone appears to deal with generalized anxiety effectively. The benzodiazepine compounds are also effective at reducing symptoms of panic and worry (Table 2).

Sialorrhea, or excessive drooling, occurs not from making too much saliva but from the slowing of the swallowing reflect the action that routinely happens [85]. Several forms of treatment range from atropine drops, Botulinum toxin, and glycopyrrolate (an oral anticholinergic) (Table 2). Constipation is a common gastrointestinal problem in PD [86]. PwP should follow a good diet and preventative maintenance (e.g., drink plenty of fluids, use dietary fiber products). Several medications can be used for treating constipation (Table 2), but Reglan, Compazine, and Phenergan should be avoided since they are dopamine-blocking compounds.

Table 3 gives a list of the majority of drugs approved by the FDA that are available to treat the non-motor symptoms of PD related to dementia and psychosis, sleep disorders, cognition, orthostatic hypotension, and urinary incontinence [79,80,81,82,83]. Mild cognitive impairment that progresses to dementia is a major concern to PwP [87]. The group of acetylcholinesterase inhibitors (Donepezil, Galantamine, and Rivastigmine) are frequently used in treating PwP for cognitive impairment and dementia. If PwP begin experiencing visual hallucinations or delusions, in addition to other symptoms of psychosis [88], besides a detailed assessment by the healthcare team, Pimavanserin, Clozapine, and Quetiapine have been used in PD. An interesting side effect of Clozapine is an anti-tremor effect in PD [89,90].

There are many different forms of sleep disorders in PD [91]. Sleeping disorders can range from poor tremor control and reduced bed mobility, restless leg syndrome, insomnia and rapid-eye-movement (REM)-sleep behavior (RBD). A related and frequent coexisting problem in PD is obstructive sleep apnea, which is evaluated by an overnight evaluation in a sleep laboratory. Combined with sleeping disorders is a widespread occurrence of excessive daytime sleepiness. The healthcare team will carefully assess treatment strategies to describe the sleeping history of the PD patient.

Orthostatic hypotension is a significant decrease in blood pressure when someone rises from a sitting or lying position to a standing position [92]. Orthostatic hypotension can be enhanced by dopamine agonists and carbidopa/levodopa [93]. As mentioned above for sleep disorders, a careful history by the healthcare team may decide to lower the motor-symptom medication, change in lifestyle (e.g., drink more fluids, wear support stockings) before moving on to medications that increase blood pressure. The loss of bladder control (urinary incontinence) and urinary urgency/frequency are relatively common issues in PD [94,95]. There are several types of medications that help regain control of the bladder. However, the healthcare team needs to fully assess this non-motor PD problem before using medication to relax the bladder (e.g., exclude urinary tract infection and enlarged prostate in men).

### 2.4. Restorative Options for Treating PD

One key goal for any PD treatment strategy is to achieve some form of neuroprotection. Of the therapeutic compounds featured in Table 1, there is evidence of neuroprotection from only Selegiline (Eldepryl, Zelapar) and Rasagiline (Azilect) in cell culture and rodent models of PD [96]. In the DATATOP (Deprenyl and Tocopherol Antioxidative Therapy of Parkinsonism) study, researchers showed a significant but small symptomatic benefit for Selegine; however, it was not classified as truly neuroprotective [97,98]. Azilect was studied in large follow-up clinical studies, named ADAGIO (Attenuation of Disease progression with Azilect Given Once-daily) [99] and TEMPO (Rasagiline in Early Monotherapy for Parkinson’s Disease Outpatients) [100]. Ultimately, the FDA did not give these studies approval to label Rasagiline as neuroprotective. Thus, the long-term neuroprotective effect for Selegiline and Rasagiline remains an open question.

The potential for exercise to be neuroprotective in PD has been widely studied [10,12,22,23,24,25,26,27,28,29,30,31,32,33,34,35,36,37,38,39,40,41,42]. There is emerging evidence that neuroinflammation contributes to the progression of PD [101,102]. Exercise is believed to help mitigate this pathogenic effect and so has been studied as a potential therapy. Exercise studies of a mouse model of PD found that exercise preserved remaining dopaminergic neurons and was associated with both an increase in brain-derived neurotropic factors and a reduction of pro-inflammatory markers [32,103,104,105]. Collectively, the animal results have shown that strenuous aerobic exercise is neuroprotective in PD through the inhibition of alpha-synuclein accumulation. Furthermore, the animal studies have suggested strenuous aerobic exercise promotes both anti-oxidation and anti-inflammatory properties not only in the brain but systemically. Human studies have shown that moderate exercise improves QoL in PwP and that strenuous aerobic exercise likely has a neuroprotective effect in PD [22,25,46,106,107].

Stress and mindfulness studies are beginning to demonstrate that being mindful can substantially benefit PwP [108,109]. Exercise studies have also been performed to address non-motor symptoms in PD [10,11,12,35,50]. Aerobic exercise continues to be a key treatment in potentially being neuroprotective in PD [110,111]; however, as mentioned before, there are several exercise routines that improve QoL in PD [8,16,22,24,50,51,52].

There is a growing interest in trying to understand the interplay between muscle and bone factors synthesized in response to exercise [112,113,114]. Exercise has been found to promote substances that have been termed ‘exerkines’, shown to influence homeostasis [115,116]. One recently described exerkine is interleukin-13 (IL-13), which is produced in mouse skeletal tissue and increases with exercise [117,118]. The impact of exercise led to the increased synthesis of IL-13 that promoted endurance in the animal. This and other related findings suggest these circulating bioactive substances may cross the blood-brain barrier and possibly offer protection from PD and other neurodegenerative disorders.

### 2.5. Maintenance Options for Treating PD

With the exception of strenuous aerobic exercise, there is no known therapy or drug treatment regimen found to slow down PD progression. In this absence of many neuroprotective options, numerous PwP have turned to a CAM and integrative medicine strategy [16,17,18,19,20,21]. Such strategies aim to preserve remaining dopaminergic neurons with the added potential to reduce neuroinflammation, maintaining PD from progressing further. Discussed below are the potential benefit of various compounds in PD maintenance.

There is renewed interest in understanding how vitamin D_3_ impacts the progression of PD [119,120,121,122]. PwP with deficiencies in vitamin D_3_ have impaired motor function and increased disease severity [120,122]. PwP with higher levels of vitamin D_3_ had better cognitive performance with improved verbal fluency and verbal memory [121]. Magnesium l-threonate has been shown to cross the blood-brain barrier and may assist dopaminergic cell survival [123,124,125]. Vitamin B_1_ has been found to generally support cognition and is vital for healthy nerves [126,127,128]. Taurine has been shown to down-regulate pro-inflammatory microglial cells in a mouse model of PD [129,130,131].

**Figure 3:**
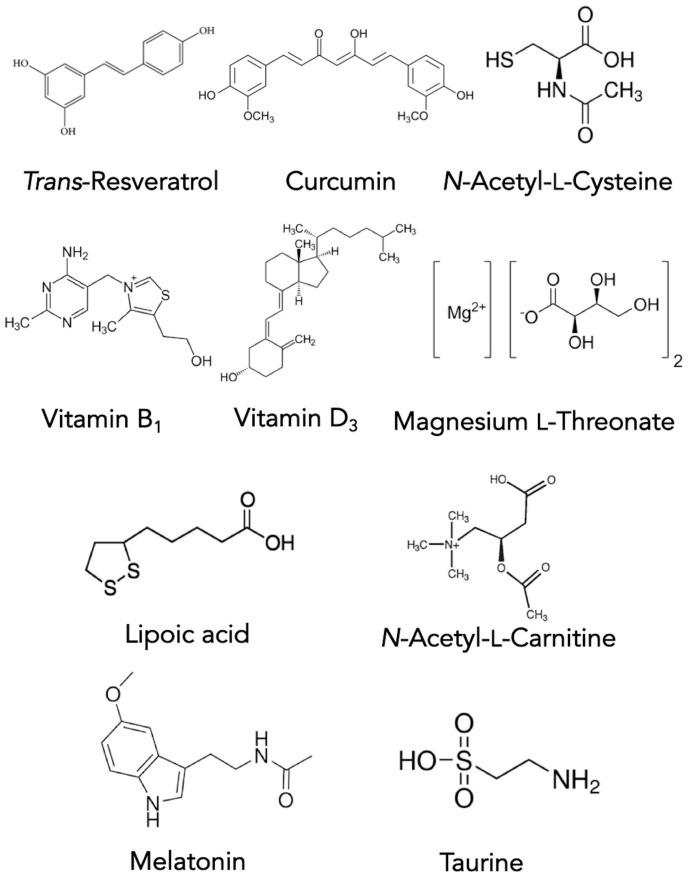
Structures of the CAM maintenance compounds used for PD treatment.

The ultimate goal of CAM therapy is to help maintain the health of remaining dopaminergic cells healthy in PwP. Curcumin is a well-known polyphenol with antioxidant properties [132,133,134]. Curcumin both inhibits NFkB and MAPK and prevents free radical damage. Alpha lipoic acid and acetyl-l-carnitine have been shown to reverse and partially restore mitochondrial function and reduce oxidative vulnerability in experiments using aging mice [135,136,137,138]. This suggests that these compounds may offer benefit to the aging population. *N*-acetyl-cysteine (NAC), one of the building blocks of the important antioxidant glutathione, crosses the blood-brain barrier and reduces oxidative stress. However, NAC also contributes to the reducing potential of the brain, as NAC is thought to be the rate-limiting factor in the production of glutathione [139,140]. Trans-resveratrol is a potent antioxidant capable of reducing free radicals in the environment of the brain [141,142]. Finally, melatonin regulates the sleep-wake cycle and also has been shown to protect mitochondria [143]. Collectively, these compounds may help preserve remaining dopaminergic neurons in the PD brain. Their molecular structures are depicted in Figure 3.

### 2.6. Surgery Options for Treating PD

Deep brain stimulation (DBS) is the surgery used for PD treatment typically in PwP suffering long-term complications from carbidopa/levodopa therapy. Currently, DBS surgery for PD is considered reversible, as brain tissue is not destroyed, stimulation can be adjusted as the disease progresses, and DBS can be performed bilaterally without significant increase in adverse events [144,145,146,147,148].

Refinement of the DBS technique has led to increased understanding of the connection between basal ganglia and PD pathophysiology [149,150,151]. Typically, DBS targets three structures in the brain, specifically the thalamus, globus pallidus, and subthalamic nucleus [144,145,146,147,148]. PwP are recommended for DBS surgery because of motor complications that do not respond further to medical therapy [5]. It should be noted that the PD patient still has a good response to carbidopa/levodopa, but this is complicated by excessive dyskinesia.

## 3. PD Clinical Trials in Progress and Novel (and Emerging) PD Therapies

MacFarthing et al. recently reviewed the current number of clinical trial pipelines with a goal of either stopping, slowing, or reversing PD [152]. They found 145 registered and ongoing clinical trials targeting PD spread out among Phase 1, Phase 2, and Phase 3. Interestingly, 57 clinical trials are aimed at long-term disease modifying therapies while 88 clinical trials are centered on symptomatic relief. Furthermore, 50 of these clinical trials are testing repurposed therapies [152]. One recent approved repurposed therapy is a new inhaled version of levodopa (Inbrija), approved by the FDA in December 2018. Two such compounds considered disease modifying therapy include Exenatide and glial cell-derived neurotrophic factor (GDNF). Exenatide (currently there are five clinical trials) is a glucagon-like peptide-1 receptor agonist that reduces glucose in type 2 diabetes; however, early results suggest that Exenatide is neuroprotective in PD [152]. Based on neurorestorative and neuroprotective effects in animal models of PD, there are currently three clinical trial evaluating the influence of GDNF in PD [152]. This detailed review of on-going clinical trials by MacFarthing et al. offers some encouraging results, describing a wide range of therapeutic approaches in multiple phases of clinical testing and evaluation in PD [152].

The transplantation of neuronal cells (fetal midbrain tissue) into PD patients’ brains has led to various stem cell therapy forms [153,154]. There are currently two clinical trials using embryonic stem cells (ESC) therapy for PD (in Australia and China). A clinical trial was recently started in Japan using induced pluripotent stem cell (iPSC)-derived dopaminergic neurons [155]. Cell-based therapy offers a chance to renew and replace dopaminergic neurons in PD; therefore, there is much interest in these necessary clinical trials’ safety and outcome.

The red-light-helmet for treating PD is under investigation [156,157,158], which uses a helmet lined with light-emitting diodes (LEDs) of wavelengths across the red to near-infrared range (i.e., 670, 810, and 850 nm) with or without an intranasal LED device (660 nm). Preliminary results are promising regarding improved symptoms of the tested PwP [156,157,158].

## 4. Conclusions

PD is a complicated and chronic disorder featuring the development and progression of both motor and non-motor defects. This review lays out a five-part management strategy for PD. One of the confounding aspects of PD is the wide heterogeneity of patient presentations; one PwP may have had the disorder for many years with minimal symptoms while another PwP may be newly diagnosed and experiencing significant disease progression. Different disease presentations demand different management strategies. Hopefully, this review may aid PwP and their care teams in developing comprehensive and personalized management plans. In particular, it is hoped the overview provided here might help inform how not only to manage PD symptoms but also to improve QoL for PwP.

## Figures and Tables

**Table 1:** Therapeutic Options for Treating the Motor Symptoms of Parkinson’s Disease.

Type Compound	Drug Name	Brand Name	Additional Description
Dopamine replacement	Carbidopa/Levodopa	Sinemet IR, Sinemet CR, Rytary, Duopa	Sustained-release capsules
		Rytary	
		Duopa	Enteral suspension in jejunum
		Inbrija	Enhaled powder of Levodopa alone (no carbidopa)
Dopamine agonist (DA)	Apomorphine	Apokyn	Nonergoline receptor
		Kynmobi	antagonist
	Pramipexole	Mirapex	Stimulates D2 dopamine receptors
		Mirapex ER	
	Ropinirole	Requip	Stimulates D2 dopamine receptor
		Requip XL	
	Rotigotine	Neupro	Transdermal patch
MAO-B Inhibitors	Selegiline	Eldepryl	Blocks the breakdown of dopamine
		Zelapar	
	Rasagiline	Azilect	
	Safinamide	Xadago	
COMT Inhibitors	Opicapone	Ongentys	Catechol-*O*-methyl- transferase (COMT) inhibitors make more levodopa available for transport across the BBB
	Entacapone	Comtan	
	Carbidopa, levodopa, and Entacapone	Stalevo	
	Tolcapone	Tasmar	
Anticholinergic	Trihexyphenidyl	Cogentin	Provides relief from tremor
	Benztropine mesylate		
Anti-influenza drug	Amantadine	Symmetrel	Provides relief for most motor symptoms, but effect is short-term.
		Gocovri	
		Symadine	

**Table 2:** Therapeutic Options for Treating Non-Motor Symptoms of Parkinson’s Disease: Depression and Anxiety, Drooling, and Gastrointestinal Problems.

Symptom/Type Compound	Drug Name	Brand Name	Additional Description
**Depression and Anxiety**			
Benzodiazepine	Alprazolam	Xanax, Xanax XR, Niravam	Anxiety and panic
	Clonazepam	Klonopin	Anxiety and panic
	Diazepam	Valium	Anxiety and panic
	Lorazepam	Ativan	Anxiety and panic
Selective Serotonin Reuptake Inhibitors (SSRI)	Fluoxetine	Prozac	Depression, panic, anxiety
	Sertraline	Zoloft	Depression, panic, anxiety
Serotonin/Norepinephrine Reuptake Inhibitors (SNRI)	Duloxetine	Cymbalta	Depression and anxiety
	Desvenlafaxin	Pristiq	Depression and anxiety
	Milnacipran	Savella	Depression and anxiety
	Venlafaxine	Mirapex	Depression and anxiety
		Effexor/Effexor XR	
Tricylic compounds	Amitrytiline	Elavil	Depression, anxiety
	Imipramine	Tofranil/Tofranil PM	Depression, anxiety
	Nortriptyline	Pamelor	Depression, anxiety
Additional anti-anxiety	Buspirone	BuSpar	General anxiety
	Propanolol	Inderal/Inderal LA	Panic attack/anxiety
	Quetiapine	Seroquel	Depression/anxiety
	Trazodone	Desyrel, Oleptro	Depression/anxiety
Other anti-depressants	Bupropion	Wellbutrin SR/XL/SR/XL	Depression
		Zyban	
	Mirtazapine	Remeron/SolTab	Depression
**Excessive Drooling**	Atropine drops		Unwarranted drooling
	Botulinum toxin A	Xeomin	Unwarranted drooling
	Botulinum toxin B	Myobloc	Unwarranted drooling
	Glycopyrrolate		Unwarranted drooling
	Scopolamine patch		Unwarranted drooling
**Gastrointestinal Problems**			
Constipation	Lubiprostone	Amitiza	Constipation
	Polyethylenene glycol	MiraLax	Constipation
Nausea and Vomiting	Ondansetron	Zofran	Nausea, vomiting
	Trimethobenzamide	Tigan	Nausea, vomiting

**Table 3:** Therapeutic Options for Treating Non-Motor Symptoms of Parkinson’s Disease: Dementia and Psychosis, Sleep Disorders, Cognition, Orthostatic Hypotension, and Urinary Incontinence.

Symptom/Type Compound	Drug Name	Brand Name	Additional Description
**Dementia**			
Acetylcholinesterase Inhibitors	Donepezil	Aricept	Dementia
	Galantamine	Razadyne/ER	Dementia
	Rivastigmine	Exelon, Exelon Patch	Dementia
**Psychosis**			
	Clozapine	Clozaril, FazoClo	Hallucinations/Psychosis
	Pimavanserin	Nuplazid	Hallucinations/Delusions
	Quetiapine	Seroquel, Seroquel SR	Hallucinations/Psychosis
**Sleeping Disorders**			
	Amitriptyline	Elavil	Insomnia
	Clonazepam	Klonopin	REM Sleep Behavior Disorder
	Doxepin	Silenor	Insomnia
	Eszopicione	Lunestra	Insomnia
	Melatonin		Insomnia
	Mirtazapine	Remeron	Insomnia
	Trazadone	Desyrel	Insomnia
**Cognition and Daytime Sleepiness**			
	Methylphenidate	Concerta	Unable to focus, overly sleepy during the day, fatigue
		Daytrana Patch	
		Metadate CD	
		Methylin	
		Ritalin, Ritalin LA/SRF	
	Memantine	Namenda	PD related dementia
	Modafinil	Provigil	Unable to focus, sleepy during the day
**Orthostatic Hypotension**			
	Fludrocortisone	Florinef	Neurogenic Orthostatic Hypotension
	Pyridostigmine	Mestinon	
	Droxidopa	Northera	
**Urinary Incontinence**			
Anticholinergics	Darifenacin	Enoblex	Overactive bladder
	Oxybutynin	Ditropan/XL, Glenique, Oxytrol	Overactive bladder +/− Incontinence
	Solifenacin	Vesicare	Overactive bladder
	Tolterodine	Detrol/LA	Overactive bladder
Beta-3-Agonist	Mirabegron	Mybetriq	Overactive bladder
Alpha-1A blockers	Alfuzosin	Uroxatral	Overactive bladder, benign prostatic hyperplasia (BPH)
	Silodosin	Rapaflo	Overactive bladder, BPH
	Tamsulosin	Flomax	
	Terazosin		
Serotonin, Norepinephrine Reuptake Inhibitors (SNRI)	Duloxetine	Cymbalta	Urinary incontinence from stress

## Data Availability

Not Applicable.
